# Use of Shotgun Metagenomics to Assess the Microbial Diversity and Hydrocarbons Degrading Functions of Auto-Mechanic Workshops Soils Polluted with Gasoline and Diesel Fuel

**DOI:** 10.3390/microorganisms11030722

**Published:** 2023-03-10

**Authors:** Emerance Jessica Claire D’Assise Goma-Tchimbakala, Ilaria Pietrini, Joseph Goma-Tchimbakala, Stefano Paolo Corgnati

**Affiliations:** 1 Energy Center Laboratory, Department of Energy (DENERG) Politecnico di Torino, 10138 Torino, Italy; 2 Institut National de Recherche en Sciences Exactes et Naturelles (IRSEN), Brazzaville BP 2400, Congo; 3 Eni R&D, Environmental and Biological Laboratories, Eni SpA, 20097 Milan, Italy; 4 Ecole Nationale Supérieure d’Agronomie et de Foresterie, Université Marien Ngouabi, Brazzaville BP 69, Congo

**Keywords:** shotgun metagenomics, diesel/gasoline contamination, bacterial diversity, hydrocarbons pathways, bioremediation

## Abstract

Bioaugmentation is a valuable technique for oil recovery. This study investigates the composition and functions of microbial communities in gasoline- and diesel-contaminated soils of garages Matoko (SGM) and Guy et Paul (SGP) originating from auto mechanic workshops as well as the concentration of soil enzymes β-glucosidase, β-glucosaminidase, and acid phosphatase. The work aimed to evaluate the presence of petroleum-hydrocarbon-degrading bacteria for the development of foreseen bioremediation of oil-contaminated soils. Microbial diversity, as given by shotgun metagenomics, indicated the presence of 16 classes, among which Actinobacteria and Gammaproteobacteria dominated, as well as more than 50 families, including the dominant Gordoniaceae (26.63%) in SGM and Pseudomonadaceae (57.89%) in SGP. The dominant bacterial genera in the two soils were, respectively, *Gordonia* (26.7%) and *Pseudomonas* (57.9%). The exploration of the bacterial metabolic abilities using HUMANn2 allowed to detect genes and pathways involved in alkanes and aromatic hydrocarbons in the two contaminated soils. Furthermore, enzymes β-glucosidase, β-glucosaminidase, and acid phosphatase were found in high concentrations ranging between 90.27 ± 5.3 and 804.17 ± 20.5 µg pN/g soil/h, which indicated active microbial metabolism. The high diversity of microorganisms with a hydrocarbon degradation genetic package revealed that the bacteria inhabiting the two soils are likely good candidates for the bioaugmentation of oil-contaminated soils.

## 1. Introduction

Among the numerous sources of soil pollution, anthropogenic activities linked to the operations of auto mechanic workshops account for a considerable proportion. Indeed, in many countries, vehicular repair garages are located all over communities fitted out either in makeshift spaces, i.e., roadsides and streets without particular arrangement or in dedicated buildings with no consideration for environmental protection [1,2,3,4]. Therefore, without proper waste-handling systems, the extensive range of vehicular waste from maintenance activities and derived petroleum products comprising used motor oil, brake lubricants, gasoline, and diesel are usually directly dumped onto the soil [1,2,3,4,5]. Moreover, the hydrocarbon molecules constitutive of petroleum products are persistent in the environment and bioaccumulate in living organisms with harmful after-effects [6,7]. The contaminants are also subjected to leaching phenomena due to rains or domestic water that drain them underground and outside the mechanic workshop area, therefore expanding the pollution [3]. 

However, petroleum-contaminated environments constitute rich reservoirs of microbial populations with hydrocarbon-catabolic abilities. Indeed, oil contamination favors the expansion of microorganisms capable of degrading these compounds through synergetic actions and cooperative relationships [8,9]. Hence, as chronically oil-stressed areas, soils from auto mechanic workshops (SAW) represent a valuable source of microorganisms with the ability to break down hydrocarbon contaminants and therefore be used in bioremediation strategies [5,8,10,11]. In fact, hydrocarbons’ persistence in the environment and especially in soil ecosystems is still an up-to-date issue. Therefore, the remediation of contaminated areas has been globally accepted to be the most efficiently, eco-friendly, and cost-effectively tackled by the exploitation of microorganisms’ natural abilities to disrupt the contaminants’ chemical structure, possibly until their complete degradation [6,12,13]. 

An increasing focus for soil remediation and recycling is on the bioaugmentation technique. This approach consists in improving the contaminated site’s dwelling community by adding isolated and well-characterized microbial biomass capable of metabolizing the pollutants to achieve enhanced oil remediation [6,13]. In this regard, exploring the microbial communities’ composition of SAW and their activity represents a good starting point to evaluate their possible contribution towards bioaugmentation for the remediation of oil-contaminated soils. Indeed, most of the studies in the literature emphasize the pollution caused by auto mechanic workshops and the toxicity potential of dumped petroleum compounds [3,4,5], while the environmental contribution of SAWs for hydrocarbon-contaminated soils treatment is often overlooked. Only a few studies have reported the isolation of hydrocarbon-utilizing bacteria from SAWs for bioremediation purposes [14]. In particular, the metagenomes analysis of SAWs for investigating genes and pathways associated with hydrocarbon degradation has not yet been sufficiently studied. Such information is obtainable through high-throughput sequencing techniques that disclose the diversity of indigenous populations in a sampled community and their abilities via present genes and metabolic routes breakdown [15,16].

Here, the microbial communities’ composition of two SAWs contaminated with diesel and gasoline was investigated by shotgun metagenomics analysis, and their enzymatic activity was determined. Indeed, soil enzymes such as β-glucosidase, β-glucosaminidase, and acid phosphatase are sensitive to environmental disturbances. Therefore, they represent suitable biomarkers of microbial activity, considering that they are involved in carbon, nitrogen, or phosphorus cycling and organic matter degradation, which has been used to assess soil remediation efficiency [13,17].

In addition, we also sought to elucidate the presence of genes and pathways involved in hydrocarbon degradation in order to acknowledge the presence of potential hydrocarbon-oxidizing bacteria. Finally, an attempt to recover strains usable in future bioaugmentation strategies involving allochthonous microorganisms was conducted by isolating bacteria able to grow using petroleum hydrocarbons as carbon sources and producing biosurfactants. Indeed, biosurfactant molecules enhance hydrocarbon bioavailability, a critical factor for the successful degradation of these compounds [7]. The use of biosurfactants-producing bacteria is advantageous for implementing a bioaugmentation strategy. 

## 2. Materials and Methods

### 2.1. Sites Location and Soil Sampling

Two hydrocarbon-contaminated sites located at auto mechanic workshops were studied. The two auto garages in this study diverged by the oil products preferentially manipulated: gasoline in the auto garage “Matoko” and diesel fuel in the auto garage “Guy et Paul”. Soil samples collected in those sites will be called SGM (4°17′14.3″ S, 15°15′33.7″ E) and SGP (4°17′35.5″ S, 15°15′30.8″ E), respectively. The hydrocarbon-contaminated soil samples were collected at five locations in each site. The soil samples were taken in the 0–10 cm horizon using a 5 cm diameter auger. The soil was then transported to the laboratory in iceboxes. At the laboratory, the stones, debris, and roots were removed from the soil by sieving at 2 mm. Each sample was homogenized, then, 20 g of soil were put in sterilized tubes and stored at −80 °C for DNA extraction and metagenomics analysis. The remaining soils were used to analyze the physicochemical properties (Table 1).

### 2.2. DNA Extraction and Next-Generation Sequencing

Soil samples were sequenced at Mr. DNA laboratory, Texas and processed as described in [18]. Briefly, 20–50 ng of DNA extracted from 250 mg of soil samples were used to prepare the libraries with a Nextera DNA Sample preparation kit (Illumina, San Diego, CA, USA), following the manufacturer’s instructions. After adding adapters, the libraries were pooled, diluted (to 14.0 pM), and pair-end sequenced for 300 cycles using a HiSeq system (Illumina). The obtained reads (quality score >35) were assembled on Galaxy instances [19] using MEGAHIT version 1.1.3.5 (accessed in October 2021) [20]. Then, Bowtie2 version 2.3.4.3 (accessed in October 2021) [21] was used to map the reads against the assembled genomes. Subsequently, after filtration of chimeric sequences, MetaPhlAn2 v2.6.0.0 (accessed in October 2021) [22] was used to determine the taxonomic affiliation of the contigs. 

### 2.3. Annotation and Functional Profiling of the Assembled Metagenomes

The functional annotation of the communities was carried out with HUMANn2 v0.11.1.0 [23] using the contigs fasta files. Contigs were aligned using Diamond. Functions were searched against the protein database UniRef50, while pathways were computed against MetaCyc and UniPathway databases considering a 10^−5^ E-value threshold. In order to minimize as many false positives as possible, an 80% coverage threshold for alignments was considered. In addition, the metabolic capacities for hydrocarbon degradation and the particular genera involved in the process were also assessed. 

### 2.4. Diversity Assessment

Alpha and beta diversity analyses were conducted using Past 3.2.6 b software. Richness and diversity were estimated by calculating Chao1, Abundance-based, Shannon’s Index, Simpsons’ Diversity Index, and Pielou’s Evenness. 

### 2.5. Carbon and Nitrogen Cultivable Microbial Biomass and Soil Enzymes

A modified technique from [24] was employed. Soil samples were hydrated to 50% of their capacity. Then, they were pre-incubated at 30 °C for 5 days in aerobic conditions to stabilize microbial activity. After 30 min of agitation, 20 g of wet soil was extracted with 50 mL of K_2_SO_4_ 0.5 M. The obtained soil solution was filtered using Whatman n° 42 filter paper. Afterward, 10 g of residual wet soil was placed in a 100 mL Erlenmeyer and incubated for 24 h in a desiccator in the presence of 25 mL of chloroform (CHCl_3_) without adding ethanol. The experiment was conducted in triplicate. After incubation, CHCl_3_ was discarded by ventilation, and the soil was extracted with K_2_SO_4_, as described above.

The organic carbon (C) in soil extracts was measured with a DR890 colorimeter and by employing the DCO method by HACH ^TM^ after heating at 150 °C [25]. Extractable nitrogen (N) was measured by the spectrophotometric method after nitrogen mineralization in NH_4_^+^ using indophenol blue [26]. 

Microbial biomass carbon (MBC) and nitrogen (MBN) were, respectively, calculated as C and N difference of concentration between fumigated and non-fumigated extracts. Extraction efficiency factors of 0.45 and 0.54 were, respectively, used for MBC and MBN [27] according to the following formulas:

MBC (µg C/g soil) = Ec/KEc,(1)

where KEc = 0.45 with Ec = (organic C extracted from fumigated soil) − (organic carbon extracted from non-fumigated soil);

MBN (µg N/g soil) = E_N_/KE_N_, (2)

where KE_N_ = 0.54 with E_N_ = (organic N extracted from fumigated soil) − (organic N extracted from non-fumigated soil). 

### 2.6. Determination of Soil Enzymes

Soil enzyme activities have been considered as parameters to provide a biological assessment of soil function, and several soil enzyme activities have been proposed for evaluating and monitoring the remediation of hydrocarbon-contaminated soils [28]. This study considered the following enzymes: β-glucosidase, β-glucosaminidase, and acid phosphatase. 

Soil β-glucosidase was measured by adding a modified universal buffer (pH 6), 0.025 M toluene, and p-nitrophenyl-β-D-glucoside solutions to the soil. Then, the samples were incubated at 37 °C for 1 h. The released p-nitrophenol was quantified with a spectrophotometer at 410 nm [29].

Soil β-glucosaminidase activity was measured according to the method described by [30]. Briefly, 4 mL of 0.1 M acetate buffer (pH 5.5) and 1 mL of 10 mM p-nitrophenyl-N-acetyl-β-D- glucosaminidase solution were added to 1 g of soil and incubated at 37 °C. After 1 h of incubation, 1 mL of 0.5 M CaCl_2_ and 4 mL of 0.5 M NaOH were added to stop the reaction. The samples were swirled and filtered through Whatman no. 2v filter paper. The color intensity of the filtrate was measured at 405 nm with a spectrophotometer. 

Soil acid phosphatase was determined by adding a modified universal buffer (pH 6.5), 0.025 M toluene, and p-nitrophenyl phosphate solutions to the soil. The samples were then incubated at 37 °C for 1 h. The released p-nitrophenol (PNP) was quantified with a spectrophotometer at 410 nm [28,29], and cycling was determined using 1 g of air-dried soil.

### 2.7. Bacterial Strains’ Isolation and Assessment of Hydrocarbon-Degradation Abilities

Bacterial strains were isolated by the agar plate cultivation technique. Three grams of each soil sample (SGM and SGP) were inoculated in 5 mL of sterile Luria Bertani (LB) liquid medium in test tubes. The tubes were vigorously shaken to homogenize the mixture and left to rest for 1 h at room temperature in sterile conditions. Then, 100 µL of the soils’ supernatants were plated on LB agar separately. The plates were incubated overnight at 37 °C to isolate the general cultivable microorganisms.

The capacity of the bacterial isolates to degrade hydrocarbons was then evaluated according to their ability to use gasoline and diesel fuel as carbon sources for their growth. The isolates were streaked on sterile minimal medium (MM) containing per 1 liter of distilled water: 0.83 g of KH_2_PO_4_; 0.29 g of KCl; 10.0 g of NaCl; 0.42 g of MgSO_4_·7H_2_O; 0.42 g of NH_4_SO_4_; 1.25 g of K_2_HPO_4_; 20 g of agar, pH 7.2) supplemented with 0.3% of diesel or gasoline separately. The plates were incubated at 37 °C for 14 days. The experiment was realized in triplicate for result accuracy. The strains were also plated on MM medium without hydrocarbons and agar plates as controls.

In addition, bacterial production of biosurfactants was estimated by an emulsification test [31]. Each isolate was grown in sterile 50 mL nutrient broth (g/L: beef extract, 1 g; yeast extract, 2 g; peptone, 5 g; sodium chloride, 5 g) for 5 days at 37 °C. In test tubes, 2 mL of the culture was mixed with an equal volume of diesel and gasoline separately (autoclaved for 15 min at 121 °C) and vortexed at high speed for 2 min. The tubes were then incubated at room temperature for 24 h. The emulsification index E_24_ was calculated as a percentage according to the formula:

E_24_ = (He/Ht) × 100, (3)

where He is the emulsification layer’s height and Ht is the mixture’s total height. The experiment was performed in triplicate. 

### 2.8. Identification of Bacterial Strains

The most interesting strains after hydrocarbon degradation assays were identified based on 16S rRNA gene sequencing. The genomic DNA was extracted using a NucleoSpin® Microbial DNA kit (Macherey-Nagel, Hœrdt, France) according to the manufacturer’s instructions. The 16S rRNA gene was then PCR-amplified with a thermal cycler (Bio-Rad, Temse, Belgium) using the universal primers fD1: 5′-AGAGTTTGATCCTGGCTCAG-3′ and rP2: 5′-ACGGCTACCTTGTTACGACTT-3′ following this program: initial activation at 95 °C for 5 min; 30 cycles of denaturation at 95 °C for 30 s, followed by annealing at 55 °C or 58 °C for 30 s and an extension step at 72 °C for 1 min 30 s; and a final extension step at 72 °C for 5 min. PCR-amplified 16S rDNA fragments were analyzed by electrophoresis using 1% (*w/v*) agarose gel and gel loading Dye 6× or Blue DNA 10× for coloration. The DNA size marker used was the 2log (10 kb, BIOKÉ, Leiden, The Netherlands). The 16S rDNA PCR-amplified fragments were purified following the manufacturer’s NucleoSpin Plasmid Easy Pure kit protocol and finally sequenced using the Sanger technique (3130 × l Genetic Analyser (Applied Biosystems, Warrington, UK). The resulting sequences were then aligned with the software Bio Numerics 7.5 (Applied Maths, Sint-Martens-Latem, Belgium) and compared with sequences contained in the Genbank database (https://blast.ncbi.nlm.nih.gov/Blast.cgi, accessed on 16 January 2023). 

The GenBank accession numbers for the 16S rRNA gene sequences of the isolates are MG890200–MG890203. 

## 3. Results

### 3.1. Microbial Community Composition

Taxonomic composition based on MetaPhlAn2 analysis showed the dominance of prokaryotic organisms in all soils, while viruses were found exclusively in the sample SGP. 

A total of 12 phyla, 16 classes, 38 orders, 82 families, 141 genera, and 199 species were detected in all samples. Microbial community structures showed the dominance of Actinobacteria in SGM (92%), while Proteobacteria (70.8%), represented mainly by the class of Gammaproteobacteria (61.6% of relative abundance), prevailed in SGP. These two phyla have been shown to be the main inhabitants in soils chronically contaminated with refined petroleum oil while also generally occurring in oil-polluted soils [32,33,34,35]. 

The diversity index and richness calculated at the genus level showed the presence of a rich and diverse microbial community with a moderate distribution (Table 2). 

**Figure 1:**
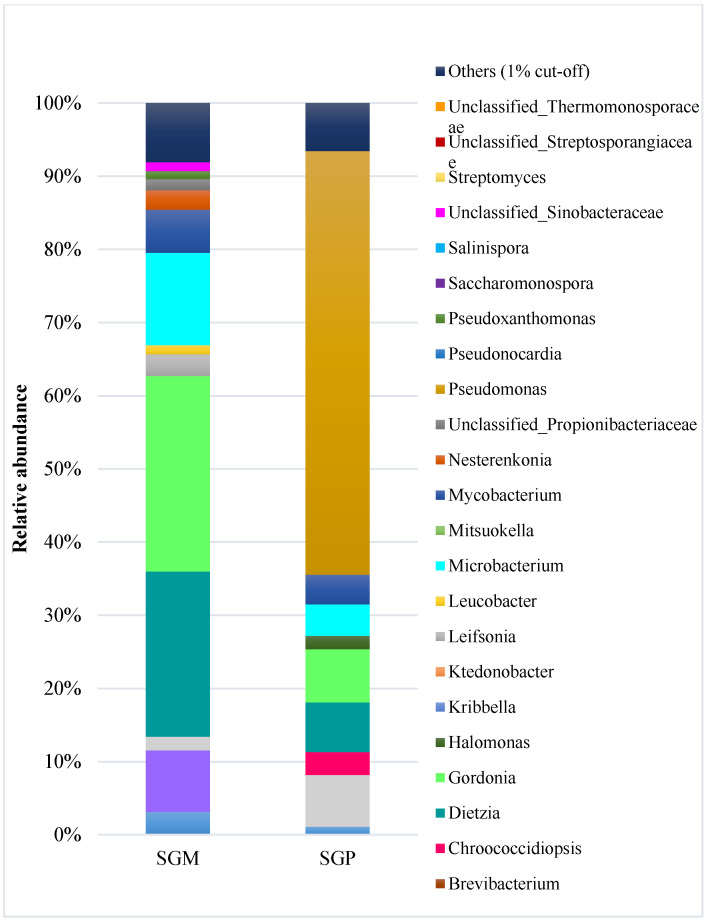
Relative abundance of the taxa at the genus level in the soil samples of the two sites SGM and SGP. Only taxa with at least 1% relative abundance are represented. The rest are grouped in the category “Others”.

The genera with the highest relative abundances were *Gordonia* (26.7%) and *Pseudomonas* (57.9%), respectively, in SGM and SGP (Figure 1). The other genera included *Dietzia* (22.6%), *Microbacterium* (12.6%), and *Mycobacterium* (5.9%) in SGM. These genera were also found in SGP with lower abundances, respectively, 6.8% (*Dietzia)*, 4.3% (*Microbacterium*), and 4.1% (*Mycobacterium*). Furthermore, the genus *Gordonia* was detected at 7.3% relative abundance in the diesel-contaminated soil. Figure 1 shows an overview of the taxonomic classification at the genus level for the two soils. 

Overall, the taxa found in the two contaminated soils are known as hydrocarbon degraders. Proteobacteria and Actinobacteria members are known for their broad catabolic abilities towards hydrocarbons. In particular, Gammaproteobacteria play a significant role in the co-metabolic processing of these compounds within the community and have been shown to be enriched following PAHs contamination [16]. Similarly, the ability of the dominant genera *Dietzia*, *Gordonia,* and *Pseudomonas* to degrade various hydrocarbons has been previously demonstrated [10,36,37,38].

### 3.2. Functional Analysis

The presence of genes and pathways associated with the degradation of hydrocarbons in members of the bacterial communities of SGM and SGP was assessed for deeper investigation of their bioremediation potential. The analysis was conducted with HUMANn2 against UniRef50, MetaCyc, and UniPathway databases with an 80% threshold for alignments. 

The results showed the presence of genes associated with aerobic degradation of alkanes in both contaminated soils. They included genes coding for alkane monooxygenase, cytochrome P450 (cytochrome P450, cytochrome P450 alkane hydroxylase, cytochrome P450 monooxygenase), alcohol dehydrogenase, and aldehyde dehydrogenase enzymes, with relative abundances of metagenomic functional genes comprising between 0.00004% and 0.05% (Table 3 and Table 4). Indeed, alkane monooxygenases oxidize *n*-alkanes to their corresponding primary alcohols. The formed alcohols are in turn oxidized by alcohol dehydrogenases that produce the corresponding alkyl aldehydes and further, carboxylic acids that enter general metabolism through the action of aldehyde dehydrogenase [39,40,41]. The alkane−1-monooxygenase enzyme, as found in this study and commonly referred to as AlkB, is encoded by the *alkB* gene widely spread in bacterial communities and is considered a biomarker for community remediation potential [35,42]. Similarly, cytochrome P450 enzymes are known to be involved in the oxidation of short-chain alkanes. As found in this study, the encoding genes are present in diverse bacterial genera, including *Dietzia*, *Gordonia*, *Mycobacterium*, and *Rhodococcus* [40,42]. Hence, the two metagenomes presented a potential for the aerobic degradation of short- and medium-chain alkanes [39,43], confirmed by the detection of an octane oxidation pathway. Other aliphatic compounds could also be degraded, as shown by the presence of an acetylene degradation pathway.

The other genes found within the two microbial communities were linked to the degradation of aromatic hydrocarbons, including biphenyl, benzene, naphthalene, toluene, and xylene (Table 3 and Table 4). Indeed, genes encoding biphenyl, catechol, or naphthalene dioxygenases and corresponding pathways were detected in the two metagenomes. As previously shown [16,44,45,46], aromatic compounds’ biodegradation likely occurs via intermediate salicylate, protocatechuate, and catechol routes. Indeed, catechol or derivatives’ formation through dihydroxylation of the benzene ring is the common initial step in the biodegradation pathways of aromatic compounds [47]. For instance, 1,2-dihydroxynaphthalene resulting from naphthalene dihydroxylation is cleaved to form salicylate, which can further be metabolized via catechol as is the case in *Pseudomonas* species [48]. Specifically, this pathway has been described in *Pseudomonas putida* G7 from naphthalene degradation to the formation of pyruvate and acetyl co-enzyme A [49]. 

The beta-ketoadipate pathway (β-KAP) was also a catabolic route found for aromatic compounds’ degradation with 0.02% and 0.05% of relative abundance, respectively, in SGM and SGP. This pathway primarily involves the conversion of the aromatic xenobiotic into catechol or protocatechuate. From there, catechol 1,2-dioxygenase and protocatechuate 3,4-dioxygenase intervene, respectively, for catechol and protocatechuate cleavage into cis, cis-muconate and β-carboxymuconate. These intermediates are further processed by enzymatic reactions to form β-ketoadipate as the fifth step of this pathway [50]. Afterwards, β-ketoadipate is converted into compounds that enter the tricarboxylic acid cycle and other general metabolic routes. Detecting both catechol 1,2-dioxygenase and protocatechuate 3,4-dioxygenase enzymes in SGP implied that the microbial community and especially *Pseudomonas*, *Gordonia*, *Dietzia*, and *Rhodococcus* affiliated genera could use either catechol or protocatechuate branch of the β-KAP for aromatic compounds degradation. Especially the existence of the beta-ketoadipate subpathway synthesizing 3-oxoadipate from 5-oxo−4,5-dihydro−2-furylacetate revealed by the functional analysis reinforced the idea of a catechol route for the biodegradation of aromatic hydrocarbons in this soil [51]. This subpathway was also found in SGM and associated with the *Pseudomonas* genus, although catechol 1,2-dioxygenase enzyme was mainly affiliated with *Gordonia* and *Dietzia*.

Overall, the contamination of the SAWs with diesel and gasoline could corroborate the diversity of the genes associated with hydrocarbon degradation found in this study. Indeed, diesel and gasoline are complex mixtures of alkanes and aromatic compounds. Hence, an adequate pool of genes encoding enzymes involved in hydrocarbon degradation is required to support the persistence of the microbial communities inhabiting the contaminated areas. For instance, [52] reported in the metagenome of a diesel-degrading consortium the occurrence of ten putative AlkB proteins belonging to the *Pseudomonas* genus, height cytochrome P450 enzymes associated with *Parvibaculum*, *Sphingobium*, and *Cupriavidus* genera as well as 83 putative ring-hydroxylating dioxygenases involved in the degradation of polycyclic aromatic hydrocarbons, such as naphthalene 1,2-dioxygenases assigned to *Sphingomonas*, *Sphingobium*, and *Bordetella* genera.

### 3.3. Soil Enzymes and Cultivable Microbial Biomass

In this study, soil biological parameters were analyzed to indicate the resilience to oil contamination of the microbial communities in SGM and SGP. 

The two soils’ microbial biomass carbon (MBC) and nitrogen (MBN) were determined. Table 5 shows high values of the two microbial biomasses. The mean values of MBC and MBN were higher in SGM (688 ± 21.4 mg C/kg soil and 186.83 ± 8.8 mg N/kg soil) than in SGP (621 ± 17.4 mg C/kg soil and 138.57 ± 10.5 mg N/kg soil). The high values of the registered concentrations suggested that contamination with diesel and gasoline might have provided the microorganisms with an important carbon source supporting their growth and metabolic activity. As previously suggested [17,28], a higher supply of hydrocarbons led to higher MBC and MBN than in the case of a lower supply of these compounds, which is in accordance with the total petroleum hydrocarbons (TPH) content of the two soils (Table 5). TPH level was higher in SGM (327.76 ± 14.19 g/kg soil) than in SGP (248.43 ± 20.21 g/kg soil). These findings suggested that the microbial populations in the two contaminated soils were likely able to cope with diesel and gasoline contamination, although natural attenuation of hydrocarbons cannot be directly acknowledged. Indeed, microbial biomass constitutes the primary biodegradation mechanism by which dissolved organic matter is broken down, and organic pollutants are removed from the soil [53,54]. 

Similarly to microbial biomasses, high values of soil enzymes β-glucosidase, β-glucosaminidase, and acid phosphatase were recorded in the two soils. Microbial extracellular enzymes are sensitive to oil-pollution-induced disturbances and are therefore considered valuable biological indicators for assessing hydrocarbon remediation. Their activity generally increases during the active phase of hydrocarbon degradation [16,55,56]. 

### 3.4. Isolation of Bacteria and Screening for Hydrocarbon Degraders

This study foresees the development of a bioaugmentation strategy for the remediation of hydrocarbon-contaminated soil, including allochthonous bacteria. In this regard, after acknowledging the bioremediation potential of the bacterial communities in the two SAWs, bacteria were isolated from the two soils, and their abilities to degrade hydrocarbons were assessed. The investigation was based on their capacity to utilize diesel and gasoline for their growth and to produce biosurfactants according to their emulsification activity. Growth on mineral medium supplemented with hydrocarbon substrates is a standard test used to directly indicate the isolates’ capability to degrade or tolerate hydrocarbons [8,14,55,56]. In addition, the emulsification test is one of the standard assays for biosurfactant production assessment. These molecules have a beneficial interest in microbial-enhanced oil recovery as they lower the surface tension at the oil–water interface, resulting in enhanced pollutants’ availability to microorganisms [7]. 

The results showed that among the 26 tested isolates, 88.5% could grow on gasoline while 61.5% could grow on diesel (data not presented).

**Figure 2:**
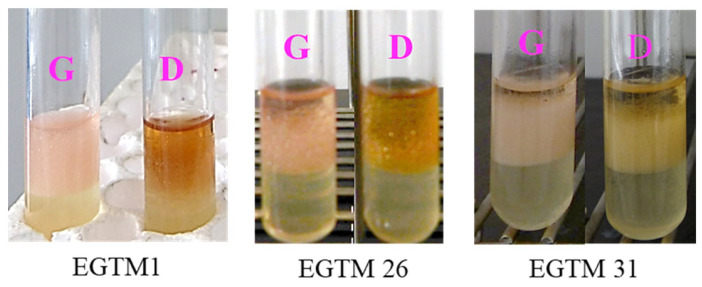
Emulsification of gasoline (G) and diesel (D) by bacterial strains. Micrococcus luteus EGTM1, Enterobacter cloacae EGTM26, and Leclercia adecarboxylata EGTM31.

The emulsification test discriminated three bacterial isolates (Figure 2), EGTM1, EGTM26, and EGTM31, with emulsification indices of 84 ± 5%, 65 ± 8, 80 ± 4% on the one hand and 53 ± 10%, 65 ± 19%, 74 ± 12% on the other hand, respectively, for gasoline and diesel. These strains were identified as *Micrococcus luteus* (EGTM1), *Enterobacter cloacae* (EGTM26), and *Leclercia adecarboxylata* (EGTM31). The species *Leclercia adecarboxylata* that arises the most interest was previously shown to degrade petroleum hydrocarbons, i.e., pyrene, catechol, naphthalene, fluorene, and fluoranthene with degradation rates ranging from 40.6% to 73.2% in 20 days [56].

Overall, it was found that the bacterial strains isolated in this study could be suitable candidates for hydrocarbon bioremediation cleanup strategies owing to their ability to use hydrocarbons for their growth and their emulsification activity.

## 4. Conclusions

In this study, shotgun metagenomics was used to trace out the composition and functions of microbial communities originating from two auto mechanic workshops soils, SGP and SGM, contaminated with diesel and gasoline. The detection of high concentrations of β glucosidase, β glucosaminidase, and acid phosphatase enzymes indicated the active mobilization of the microorganisms in the degradation of organic matter in the two soils. This supported the bacterial communities’ possible processing of petroleum hydrocarbons, since known hydrocarbon-oxidizing bacteria of the genera *Dietzia, Gordonia*, *Pseudomonas,* and *Rhodococcus* were found. The potential for hydrocarbon bioremediation was confirmed by alkanes and aromatic compounds degrading genes and pathways in the metagenomes of the two soils. Moreover, strains such as *Enterobacter cloacae*, capable of utilizing diesel and gasoline for their growth and emulsify these hydrocarbons, were isolated. This gives some room for the development of a bioaugmentation strategy using bacteria inhabiting SGP and SGM soils. 

## Figures and Tables

**Table 1:** Soils characteristics.

Soils	Clay (%)	Silt (%)	Sand (%)	Fe (%)	NH_4_ (%)	Mg (%)	C (‰)	N (‰)	P (‰)
SGM	9.5	19.94	70.52	0.25	0.17	0.09	12.5	1.0	0.03
SGP	7.03	15.33	77.64	0.33	0.15	0.07	14.2	1.2	0.02
EAR	9.77	19.14	71.10	0.37	0.18	0.10	16.2	1.7	0.04

**Table 2:** Alpha diversity in soil samples.

Alpha Diversity Index	SGM	SGP
S (number of genera)	111	81
Individuals	85	90
Simpson 1-D	0.84	0.64
Shannon H′	2.42	1.76
Evenness_e^H′/S	0.10	0.07
Equitability_J	0.51	0.40
Chao−1	111	81

**Table 3:** Main gene families, enzymes, and pathways directly or indirectly involved in hydrocarbons degradation, as well as associated genera identified in metagenomic data of SGM. Genes’ relative abundance represents the proportion of the specific gene compared to the total number of functional genes. Pathways’ relative abundance represents the proportion of the specific pathway compared to the total number of pathways.

Gene Family/Enzymes	Genus	Relative Abundance
ABC transporter	*Mycobacterium*, *Gordonia*, *Agromyces*, *Modestobacter*, *Agrococcus*, *Isoptericola*, *Cellulomonas*, *Janibacter*, *Microbacterium*, *Dietzia*, *Modestobacter*, *Rhodococcus*	0.02%
Alcohol dehydrogenase	*Gordonia*, *Mycobacterium*, *Rhodococcus*, *Dietzia*, *Paracoccus*, *Pseudomonas*, *Sphingobium*, *Geodermatophilus*, *Stenotrophomonas*	0.03%
Aldehyde dehydrogenase	*Dietzia*, *Stenotrophomonas*, *Rhodococcus*, *Mycobacterium*, *Isoptericola, Gordonia*, *Cellulomonas*, *Paracoccus*, *Janibacter*, *Xanthomonas*, *Sciscionella*, *Dechloromonas*, *Shingobium*, *Agromyces*, *Geodermatophilus*, *Agrococcus*, *Blastococcus*, *Modestobacter*, *Pseudomonas*, *Ornithinimicrobium*	0.03%
Alkane 1-monooxygenase	*Gordonia*, *Mycobacterium*, *Dietzia*, *Rhodococcus*, *Nevskia*, *Paracoccus*	0.004%
Alkane monooxygenase	*Gordonia*, *Rhodococcus*, *Mycobacterium*	0.00004%
Catechol 1,2-dioxygenase	*Gordonia*, *Dietzia*, *Geodermatophilus*	0.005%
Catechol-O-methyltransferase	*Mycobacterium*	0.0001%
Cytochrome P450	*Gordonia*, *Rhodococcus*, *Mycobacterium*, *Dietzia*, *Modestobacter*, *Blastococcus*, *Nevskia*	0.02%
Cytochrome P450 alkane hydroxylase	*Gordonia*, *Mycobacterium*, *Dietzia*	0.02%
Cytochrome P450 monooxygenase	*Gordonia*	0.0005%
Toluene efflux pump membrane transporter TtgB	*Stenotrophomonas*, *Pseudomonas*	0.0005%
Toluene tolerance protein	*Xanthomonas*, *Stenotrophomonas*	0.003%
**Pathways**	**Genus**	**Relative abundance**
Acetylene degradation	*Gordonia*, *Rhodococcus*, *Pseudomonas*, *Mycobacterium*	0.02%
Alkane degradation	*Pseudomonas*, *Nevskia*	0.00005%
Benzene degradation	*Rhodococcus*	0.0001%
Beta-ketoadipate pathway (aromatic compounds degradation via 3-oxoadipate)	*Gordonia*, *Rhodococcus*, *Pseudomonas*, *Dietzia*, *Agrococcus*, *Janibacter*, *Blastococcus*, *Xanthomonas*	0.02%
Biphenyl degradation	*Gordonia*, *Rhodococcus*, *Pseudomonas*, *Dechloromonas*	0.003%
Catechol degradation (ortho-cleavage pathway & beta; -ketoadipate)	*Not assigned*	0.003%
Naphthalene degradation	*Gordonia*, *Rhodococcus*, *Pseudomonas*, *Dechloromonas*	0.0003%
Octane oxidation	*Gordonia*, *Mycobacterium*	0.03%
P-cumate degradation	*Gordonia*, *Rhodococcus*, *Pseudomonas*, *Dechloromonas*	0.008%
Polychlorinated biphenyl degradation	*Gordonia*, *Rhodococcus*, *Pseudomonas*, *Dechloromonas*, *Nevskia*	0.02%
Protocatechuate degradation II (ortho-cleavage pathway)	*Not assigned*	0.0001%
Toluene degradation	*Dietzia*, *Gordonia*, *Rhodococcus*, *Pseudomonas*, *Dechloromonas, Paracoccus*, *Mycobacterium*, *Geodermatophilus*	0.002%
Superpathway of salicylate degradation	*Not assigned*	0.0001%

**Table 4:** Main gene families, enzymes, and pathways directly or indirectly involved in hydrocarbon degradation, as well as associated genera identified in metagenomic data of SGP. Genes’ relative abundance represents the proportion of the specific gene compared to the total number of functional genes. Pathways’ relative abundance represents the proportion of the specific pathway compared to the total number of pathways.

Gene Family/Enzymes	Genus	Relative Abundance
ABC transporter	*Gordonia, Mycobacterium, Dietzia, Pseudomonas, Agrococcus, Rhodococcus, Blastococcus, Microbacterium, Geodermatophilus*	0.01%
Alcohol dehydrogenase	*Mycobacterium, Pseudomonas, Paracoccus, Dietzia, Gordonia, Geodermatophilus, Rhodococcus, Agrococcus, Blastococcus, Chroococcidiopsis*	0.05%
Aldehyde dehydrogenase	*Pseudomonas, Thauera, Paracoccus, Mycobacterium, Dietzia, Rhodococcus, Xanthomonas, Acidiphilium, Gordonia, Blastococcus, Geodermatophilus, Chroococcidiopsis, Nevskia, Agrococcus*	0.03%
Alkane 1-monooxygenase	*Gordonia, Dietzia, Pseudomonas, Mycobacterium, Paracoccus, Nevskia*	0.002%
Alkane monooxygenase	*Gordonia*	0.0001%
Aromatic hydrocarbon degradation protein	*Pseudomonas*	0.0006%
Biphenyl-2,3-diol 1,2-dioxygenase protein	*Pseudomonas*	0.0003%
Catechol 1,2-dioxygenase	*Pseudomonas, Gordonia, Dietzia, Rhodococcus*	0.005%
Catechol oxidase	*Chroococcidiopsis*	0.0002%
Catechol-2,3-dioxygenase	*Pseudomonas*	0.0002%
Catechol-O-methyltransferase	*Mycobacterium*	0.0003%
Cytochrome P450	*Dietzia, Mycobacterium, Gordonia, Rhodococcus, Chroococcidiopsis, Blastococcus*, *Geodermatophilus, Oceanicola*	0.007%
Cytochrome P450 alkane hydroxylase	*Gordonia, Dietzia, Mycobacterium*	0.005%
Cytochrome P450 monooxygenase	*Gordonia*	0.0001%
Naphthalene 1,2-dioxygenase	*Pseudomonas*	0.002%
Protocatechuate 3,4-dioxygenase	*Pseudomonas, Gordonia*	0.002%
Salicylate hydroxylase	*Pseudomonas*	0.001%
Toluene efflux pump membrane transporter TtgB	*Pseudomonas*	0.0002%
Toluene tolerance protein	*Pseudomonas*	0.007%
**Pathways**	**Genus**	**Relative abundance**
Acetylene degradation	*Pseudomonas, Gordonia*	0.01%
Benzene degradation	*Pseudomonas*	0.001%
Beta-ketoadipate pathway (aromatic compounds degradation via 3-oxoadipate)	*Pseudomonas*	0.05%
Biphenyl degradation	*Pseudomonas, Gordonia, Rhodococcus, Thauera*	0.006%
Catechol degradation	*Pseudomonas*	0.01%
Chlorosalicylate degradation	*Not assigned*	0.0001%
4-methylcatechol degradation (ortho cleavage)	*Not assigned*	0.002%
Meta cleavage pathway of aromatic compounds	*Not assigned*	0.0005%
Naphthalene degradation	*Pseudomonas, Gordonia, Rhodococcus, Thauera*	0.003%
Octane oxidation	*Pseudomonas, Gordonia, Mycobacterium*	0.02%
P-cumate degradation	*Pseudomonas, Gordonia, Rhodococcus, Thauera*	0.007%
Polychlorinated biphenyl degradation	*Pseudomonas, Gordonia, Thauera, Nevskia, Rhodococcus, Mycobacterium*	0.01%
Protocatechuate degradation II (ortho-cleavage pathway)	*Pseudomonas*	0.007%
Superpathway of salicylate degradation	*Pseudomonas*	0.003%
Toluene degradation	*Pseudomonas, Dietzia, Paracoccus, Gordonia, Rhodococcus, Thauera,* *Mycobacterium, Geodermatophilus,*	0.01%
Xylene degradation	*Pseudomonas*	0.001%

**Table 5:** Soil microbial biomass and enzymes.

Parameter	Unit Parameter	SGM	SGP
Microbial biomass	MBC (mgC/kg soil)	688 ± 21.4	621 ± 17.4
	MBN (mgN/kg soil)	186.83 ± 8.8	138.57 ± 10.5
Soil enzymes (µg pN/g sol/h)	β glucosidase	397.13 ± 7.6	351.3 ± 9
	β glucosaminidase	101.05 ± 1.6	90.27 ± 5.3
	Acid phosphatase	804.17 ± 20.5	677.33 ± 32.1
Hydrocarbons	TPH (g/kg soil)	327.67 ± 14.2	248.33 ± 20.2

## Data Availability

Genbank accession numbers MG890200–MG890203.
